# Analysis of photo-functional materials using momentum-resolved EELS

**DOI:** 10.1093/jmicro/dfag006

**Published:** 2026-02-13

**Authors:** Yohei K Sato

**Affiliations:** Institute of Multidisciplinary Research for Advanced Materials, Tohoku University, 2-1-1, Katahira, Aobaku, Sendai, Miyagi, 980-8577, Japan

**Keywords:** electron energy-loss spectroscopy, near-infrared light scattering, anisotropic optical property, local field correction, exchange–correlation effect, photocatalysis

## Abstract

Momentum transfer (*q*)-resolved electron energy-loss spectroscopy (*q*-EELS) is a powerful tool for analyzing photo-functional materials. The technique’s application has been demonstrated in several recent studies. This study first investigated the anisotropic plasmon oscillations in Cs-doped hexagonal WO_3_, a near-infrared (NIR) shielding material, to understand the origin of its highly efficient light-scattering properties. This revealed how plasmon energies differ along different crystallographic directions, contributing to the broad NIR absorption capabilities of the material. Second, the study measured the *q* dispersion of carrier plasmons and thus quantified interactions (exchange–correlation effect) between carrier electrons in LaB_6_ crystals, another NIR shielding filter. This analysis provides critical insights into many-body effects not captured by the ideal free-electron gas model. Finally, the spatial spread sizes of excitons in anatase TiO_2_ were determined, establishing a correlation between the exciton size and the anisotropic photocatalytic activity of anatase TiO_2_. Collectively, this research demonstrates that *q*-EELS provides unique, *q*-dependent information on electronic excitations, deepening our understanding of the properties governing the performance of advanced materials.

## Teaser

Momentum-resolved EELS (q-EELS) offers unique insights into the electronic excitations governing material performance. We analyzed anisotropic plasmons in NIR-shielding WO_3_ and LaB_6_, and correlated exciton size with photocatalytic activity in TiO_2_, demonstrating the technique's power to elucidate the origins of key photo-functional properties.

## Introduction

Efficient energy utilization requires the development of photo-functional materials, such as optically controllable materials that selectively transmit or scatter specific wavelengths, photoenergy conversion materials that transform light into electrical energy, and photocatalytic materials. These materials are often employed as submicron- to nanometer-sized particles to expand their light-receiving surface. Such nanoparticle-based systems frequently exhibit optical properties and functionalities that enhance their practical performance but cannot be predicted from their bulk counterparts. The mechanisms underlying these phenomena can be clarified through measurements targeting individual nanoparticles. Among the most powerful tools for this purpose is electron energy-loss spectroscopy (EELS) integrated with transmission electron microscopy (TEM).

EELS combined with scanning TEM (STEM) can obtain energy-loss near-edge structure spectra above 50 eV, enabling elemental analysis, chemical bonding characterization, and electronic structure analysis at atomic resolution [1–5]. However, in studies of optical properties in the visible and near-infrared (NIR) regions, the important region is the low-loss region below 50 eV. In conventional TEM–EELS, measurements below ∼3 eV are hindered by the tail intensity of the zero-loss peak arising from transmitted electrons, inhibiting access to the visible and NIR spectral ranges. The optical properties below 3 eV can instead be probed using TEM–STEM instruments equipped with a monochromator [6–9]. Recent advances in monochromator technology have expanded the range of feasible spectral measurements from the visible and NIR regions to even lower energies in the infrared region [10–19]. Further, EELS-based analysis can meet the growing demand for investigating the origins of optical properties in nanoscale photo-functional materials.

Further extending the capabilities of EELS, momentum transfer (*q*)-resolved EELS (*q*-EELS) provides access to information such as anisotropic optical properties [20, 21], plasmon *q*-dispersion relations [22–24], and electronic excitations associated with band dispersion. The combination of *q*-EELS and electron microscopy enables local measurements in defect-free regions, excluding areas affected by impurities or structural defects. To date, numerous studies have reported *q*-EELS measurements based on electron microscopy for the direct observation of plasmon dispersion; however, quantitative analysis of the dielectric function as a function of *q*, which reflects optical properties and electronic structure, has rarely been explored.

This review highlights recent applications of *q*-EELS to photo-functional materials reported by our research group. A theoretical framework of dielectric functions within the free electron gas model is followed by an overview of the instrumentation, measurement procedures and analysis methods using *q*-EELS. The subsequent section presents the *q*-EELS results of Cs-doped hexagonal WO_3_ (Cs_0.33_WO_3_; CWO) [25, 26], a well-known NIR-light-scattering filter material. The exchange–correlation (XC) effects among carrier electrons are then quantitatively derived from plasmon *q* dispersion measurements of LaB_6_ crystals [27], which also scatter NIR waves. Finally, a *q*-EELS study on anatase TiO_2_ evaluates the real-space extent of excitons and correlates it with photocatalytic functionality [28]. Together, these examples demonstrate *q*-EELS as a powerful tool for probing the optical properties of photo-functional materials.

## Methods

**Fig. 1.**
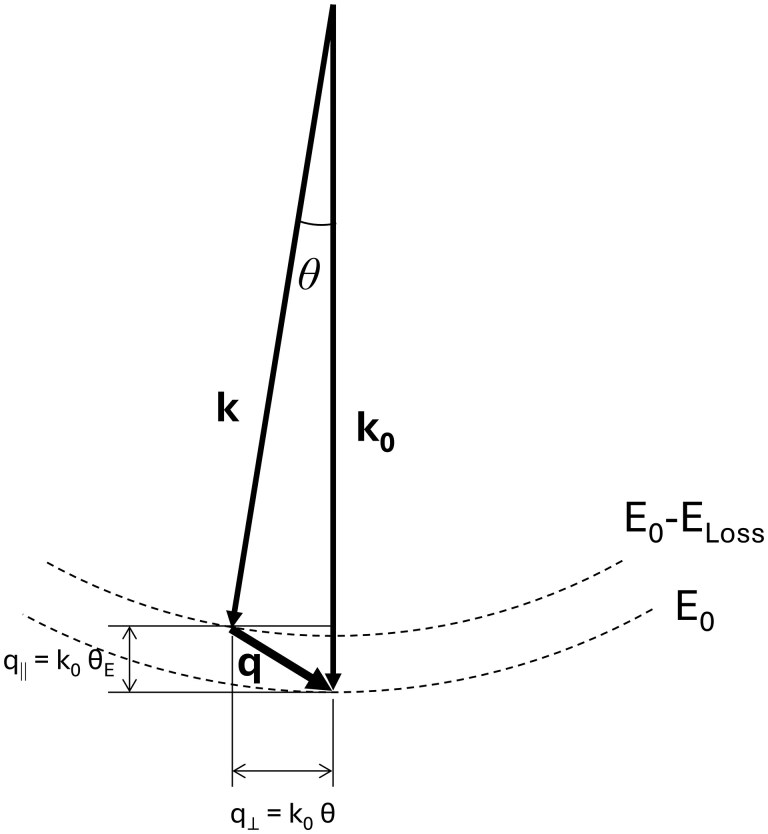
Wavenumber vectors of incident and inelastically scattered electrons (**k_0_** and **k**, respectively) and momentum transfer vector *q*.

In some inelastic scattering processes of highly accelerated electron beams, momentum is transferred to electrons in the solid. A scattered electron is deflected at angle *θ* with respect to the incident direction, and the difference between the incident wave vector ***k_0_*** and the scattered wave vector **k**, defined as ***q***  *=*  ***k****_0_ −*  ***k***, is referred to as the *momentum transfer vector* (Fig. 1). As formulated below, ***q*** is composed of a parallel component *q*_‖|_ and a perpendicular component *q*_⊥_ of the incident electron *k*_0_:


(1) $$q^{2}=q_{⊥}^{2}+q_{∥}^{2}=k_{0}^{2}(\theta^{2}+\theta_{E}^{2}).$$

The characteristic scattering angle *θ*_E_ = *E*_loss_/2*E*_0_ correlates with the localization of energy-loss events. In the NIR region where the energy loss is extremely small, *q*_⊥_ = *k*_0_*θ*_E_ ≪ 1, so *q* is approximately equal to *q*_⊥_ = *k*_0_*θ*.

From the scattering cross-section that accounts for inelastic scattering processes involving momentum transfer [29], the transition matrix element between an initial state *i* and a final state *f* can be expressed as


(2) M∝⟨f|exp⁡(iq·r)|i⟩.

The integrand of this matrix element can be expanded as


(3) $$\exp\left(iq\cdot r\right)=1-iq\cdot r+\frac{1}{2}{\left(q\cdot r\right)}^{2}-\cdots \;$$

The first term of Eq. (3) vanishes because *i* and *f* are orthogonal. When *q* is sufficiently small, Eq. (3) is dominated by the second term corresponding to dipole transitions, and the electronic excitations are analogous to optical transitions. Under the dipole approximation, the direction of vector ***q*** corresponds to the polarization direction of light. Thus, by controlling the ***q*** direction, one can obtain information equivalent to polarization-dependent absorption measurements in an anisotropic material. As *q* increases, higher-order multipole transitions that are forbidden in photoexcitation must be considered.

The initial and final states of inelastic scattering are expressed as charge-density fluctuations within the solid [30]. The excitation processes are primarily governed by longitudinal charge-density oscillations (Plasmon). Consequently, unlike optical absorption spectra, the observed EELS spectra are proportional to the loss function. The inelastic scattering cross-section depends on the scattering angle as follows:


(4) ∂σ2∂Ω∂E=12π2a0E01θ2+θE2Im-1ε(q, ω),

where *a_0_* is the Bohr radius. The term Im[−1/ε(***q****, ω*)] represents the loss function, and $\varepsilon (q,\;\omega )=\varepsilon_{1}(q,\;\omega )+i\varepsilon_{2}(q,\;\omega )$ is the complex dielectric function, which reflects the optical/dielectric properties of the material as functions of frequency and momentum transfer.

Electron energy-loss spectroscopy measurements enable direct observation of plasmons, which can be described by the random phase approximation (RPA) within the free electron gas (FEG) model. The FEG model approximates the interaction between free electrons as a mean field and adopts the following Lindhard dielectric function [31]:


(5) $$\varepsilon_{\mathrm{RPA}}(q,\omega )=\varepsilon_{L}(q,\omega )=\varepsilon_{\infty }-v_{q}\chi^{0}(q,\omega ),\;$$

(6) $$\chi^{0}(q,\omega )=\frac{2e^{2}}{V}\sum_{k}\frac{f(E_{k})-f(E_{k+q})}{ћ\omega +i\delta -E_{k}+E_{k+q}}.$$

Here, $v_{q}=4\pi /q^{2}$ is the Fourier transform of the Coulomb potential. $f(E_{k})$ is the Fermi-Dirac distribution function. *E_k_* is the energy of free electron with the effective mass m* at the Bloch wave’s wave number *k* state within a solid, $E_{k}=E(k)={\left(ћk\right)}^{2}/2m^{*}$. ε_∞_ is the background dielectric constant, which accounts for dielectric screening contributions from interband transitions at energies above the plasmon energy [32]. Using the above equation under the plasma resonance condition ε_RPA_(**q**, ω) ∼0, the plasmon dispersion relation is expressed as


(7) $$E_{P}(q)=E_{P}(0)+\frac{{ћ}^{2}}{m^{*}}\alpha \;q^{2}+\;O\;q^{4}\cdots ,$$

(8) $$\alpha =\alpha_{\mathrm{RPA}}=\frac{3}{5}\frac{E_{F}}{E_{P}(0)}.\;$$

Here,


(9) $$E_{P}(0)=ћ\sqrt{\frac{e^{2}N}{\varepsilon_{0}m^{*}}},$$

is the plasmon energy at *q *= 0, where *e* is the elementary charge, *N* is the free electron density, and m* is the electron effective mass. When plasmons arise from the carrier electrons in electron-doped semiconductors, the dielectric constant *ε*_0_ should be replaced by the background dielectric constant ε_∞_. Note that the plasmon energy is proportional to *q*^2^. A plasmon with finite momentum corresponds to a longitudinal charge-density oscillation with a wavelength proportional to 1/*q* along the ***q*** direction. As *q* increases, the spacing between the charge-density modulations becomes narrower and the electric field gradient steepens. Consequently, the driving force acting on free electrons is enhanced and the plasmon energy increases accordingly. The *q^2^* dispersion coefficient of the plasmon energy depends on the Fermi energy and effective mass of the metallic band structure. In metals with anisotropic crystal structures, the effective mass varies with crystallographic direction, so both the plasmon energy and its dispersion relation are anisotropic. In other words, by analyzing the *q*-dispersion relation of plasmons, we can discuss the electronic properties in metallic materials in terms of the FEG model.

## Experimental setup of *q*-EELS measurements

**Fig. 2.**
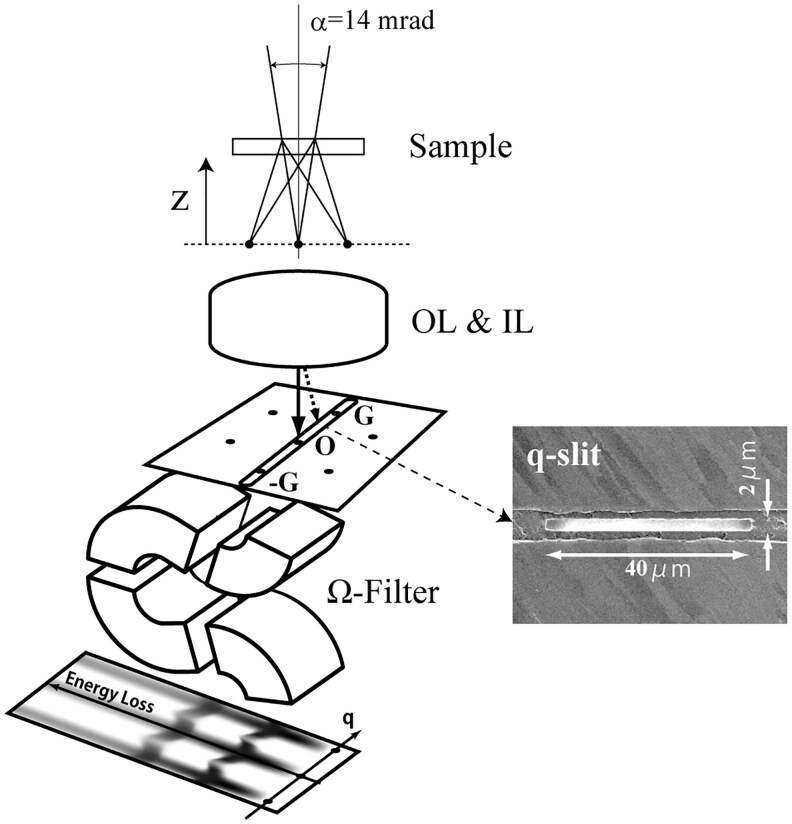
Schematic of *q*-EELS using an omega filter. Reprint from [25] with the permission of AIP Publishing.

Figure 2 is a schematic of *q*-EELS measurements using monochromator TEM with an omega filter, acquired by our group [6, 27] at 60–100 kV. The electron irradiation beam of *q*-EELS is a half convergence angle of ∼7 mrad and an electron beam probe current of approximately 3–5 pA. An electron diffraction pattern was formed at the usual specimen position by shifting the specimen upward along the z-direction. The electron illuminated specimen area was usually ∼0.2 µm in diameter. The diffraction pattern was transferred to the object plane of the omega filter through the objective lens and intermediate lenses. A part of the diffraction pattern was selected by inserting a rectangular slit in the plane (Fig. 2). Three kinds of *q*-selective slits with different slit sizes (40 μm × 2 μm, 50 μm × 1.5 μm and 50 μm × 1.0 μm) are available in the system. Each *q*-selective slit was fabricated from 10 µm-thick Mo foil using a focused ion beam device. The longer edge of the slit was set perpendicular to the energy-dispersion direction of the omega filter. The TEM specimens were mounted in a rotational holder, which can direct a specific crystal orientation parallel to the longer edge of the *q*-selection slit. The two-dimensional intensity distribution of the *E–q* map was recorded on an imaging plate (IP). The exposure time of the *E–q* map was usually set to 3–6 min per one exposure, and they were summed for 5–20 exposures.

**Fig. 3.**
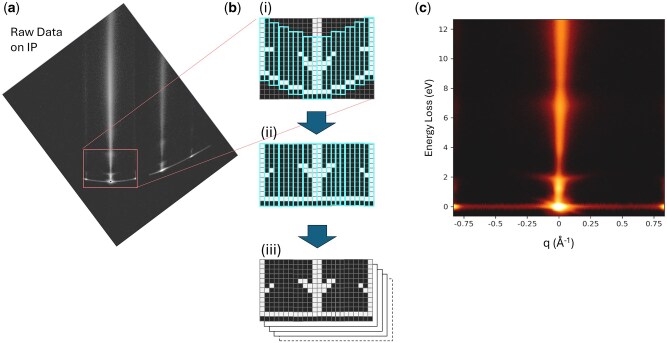
Raw data of E–q map on the imaging plate (IP). (b) Correction procedure for the raw E-q data ((i)∼(iii)). (c) Corrected E-q map.

Figure 3 illustrates the procedure from raw data acquisition on the IP to generation of the *E–q* map. The IP records the intensity data over 3760 × 3000 pixels at a pixel size of 25 μm. The raw *E–q* map data recorded on the IP exhibit a curved distortion of the quasi-elastic scattering intensity at zero energy loss (Fig. 3a). This distortion, caused by aberrations of the omega filter, is corrected by an in-house-developed image correction code in Python. The correction procedure is outlined in Fig. 3b. First, the intensity data on the IP are divided into one-pixel-wide segments. The intensity distribution along the vertical direction of each segment corresponds to the EELS spectrum. The pixel position *P*_max_ of maximum intensity within each blue frame in Fig. 2b (i) is identified as the zero-energy-loss positions and the blue frames are rearranged so that the zero-energy-loss positions are horizontally aligned. Pixels are then reordered accordingly to construct the *E–q* map (Fig. 3b (ii)). To improve the signal-to-noise ratio, multiple *E*–*q* maps are recorded on separate IPs and integrated according to the number of exposures (Fig. 3b (iii)). This procedure obtains the final *E–q* map. The *q*-resolution is evaluated from the transmitted intensity width along the *q* direction, and the horizontal axis is calibrated by referencing diffraction intensities with known indices within the *q*-selecting slit.

## Results

### Analysis of anisotropic plasma oscillation in hexagonal CWO crystals and nanoparticles

Tungsten bronzes, such as Na_x_WO_3_ and CWO, are well known as NIR light-shielding materials [8, 33–38], because the concentration of charge carriers can be tuned by adjusting the amount of doped metal ions, thereby enabling control of the plasmon energy in the NIR region. The x dependence of the plasmon energy in Na_x_WO_3_ can be directly observed by EELS measurements [39]. Furthermore, *q*-resolved EELS measurements of Na_x_WO_3_ have been reported, in which the effective mass and dielectric shielding for the carrier electron of Na_x_WO_3_ were evaluated from the *q* dependence of the plasmon energy [40]. Since Na_x_WO_3_ has a cubic crystal structure, the *q*-direction dependence of the plasmon energy is expected to be essentially isotropic. In contrast, Cs^+^ ions, which have a larger ionic radius than Na^+^, require larger interstitial sites than those in cubic WO_3_, and are therefore stabilized in a hexagonal crystal structure. CWO nanoparticles are especially effective as NIR shielding filters because they photo-scatter over a wide NIR energy range, thus achieving remarkably high scattering efficiency [33]. The CWO crystal is milled into nanoparticles and NIR scattering occurs via coupling of resonant excitation surface dipole plasmons with NIR electromagnetic waves. Although the optical scattering behavior of nanoparticles can be generally predicted using Mie theory [41] based on bulk dielectric functions, the scattering spectra of CWO nanoparticles considerably deviate from bulk predictions. The mechanism by which CWO nanoparticles scatter wide-range NIR light remains unresolved, warranting a more detailed investigation of their dielectric properties. CWO crystals possess an anisotropic hexagonal structure, which should affect their dielectric and optical anisotropies.

Hussain *et al.* [42] performed polarized reflectance measurements on CWO crystals and observed that the energy position of the steep reflectance drop (plasma edge) associated with plasmon excitation depends on the orientation of the polarization vector relative to the crystallographic *c*-axis. Kim *et al.* [43] demonstrated that CWO nanocrystals exhibit two localized surface plasmon resonance peaks in systems where the nanocrystal shape is varied from disks to rods, and that these features originate not only from differences in the nanocrystal aspect ratio but also from the anisotropic dielectric function inherent to CWO nanocrystals. Thus, carrier-electron plasmons in CWO are known to exhibit anisotropic characteristics governed by the crystal structure; however, previous studies have primarily evaluated the properties based on optical properties. The use of *q*-EELS measurements is expected to provide a more direct and deeper understanding of such plasmon anisotropy at the level of the electronic structure.

**Fig. 4.**
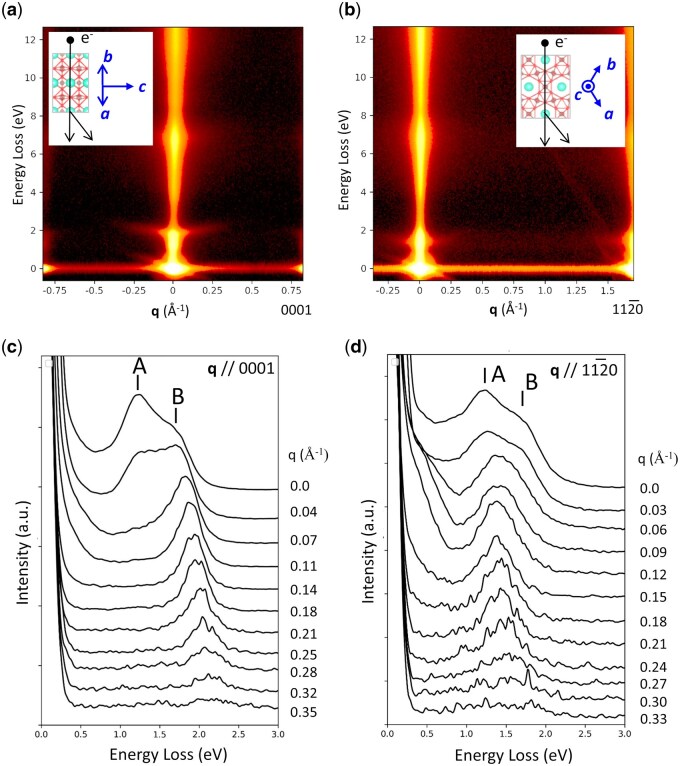
*E–q* maps of CWO crystals along (a) ***q*** // 0001 and (b) ***q*** // 112–0; *q* dependence of the EELS spectra along (c) 0001 and (d) 112–0. Reprint from [25] with the permission of AIP Publishing.

Figure 4a and b shows the *E–q* maps obtained from CWO crystals for ***q*** // 0001 (along the *c*-axis) and ***q*** // 1120 (within the *ab*-plane), respectively. The intensity observed in the 5–8 eV range originates from interband transitions from the O 2p valence band to the W 5d conduction band. The spectral intensity around 1–2 eV corresponds to plasmon excitations of the carrier electrons. The *q* dispersion of the plasmon peak appears around 2 eV in the ***q*** // 0001 *E–q* map and around the 1–2 eV range in the ***q*** // 112–0 *E–q* map. Panels (c) and (d) of Fig. 4 display the spectral profiles extracted from the 0–3 eV region of maps (a) and (b), respectively. Along both crystallographic directions, two peaks are resolved at 1.2 and 1.8 eV for *q *< 0.04 Å^−1^. As *q* increases along ***q*** // 0001, the low-energy peak A is suppressed while the high-energy peak B enhances. In contrast, increasing *q* along ***q*** // 112–0 diminishes peak B while peak A becomes dominant. As demonstrated in these results, the plasmon energies of the carrier electrons in CWO depend on oscillations along both the *c*-axis direction and the *ab*-in-plane direction.

The plasmon energies in the CWO crystal are anisotropic because the effective masses of the carrier electrons differ between the *c*-axis and *ab*-plane directions. According to theoretical calculations of the band structure of CWO [25, 44], the conduction-band minimum consists of three bands derived from the W 5d orbitals. Along the wave vector direction parallel to the *c*-axis, the dispersion of these bands crosses the Fermi level, indicating that the bands are metallic. In contrast, the d bands along the *ab*-plane direction are almost flat, corresponding to a larger effective mass of the carrier electrons. As stated in Eq. (9), the plasmon energy is higher along the *c*-axis, where the effective mass is smaller, than along the *ab*-plane. This disparity explains the experimental observations.

In practice, the light-scattering functionality of NIR filters arises from coupling between the surface plasmons of CWO nanoparticles and NIR light. Hence, the optical properties of NIR filters could be more directly evaluated by investigating the anisotropic properties of the surface plasmons of CWO nanoparticles. However, in our *q*-EELS measurement method, the electron-beam irradiation area on the specimen has a diameter of 100 nm or larger. As a result, scattered electrons are collected not only from the individual particles but also from the supporting substrate to which the particles are attached, causing the spectral intensity attributable to the particles to be obscured. Instead, the anisotropy of the surface plasmons of the nanoparticles was evaluated using the aloof-beam method.

Electron microscopy–based measurements using the aloof-beam configuration have been reported for surface plasmons in metallic nanoparticles [45, 46], nanotriangle [47], nanowires [9] and complex nanostructured materials [48]. In particular, distinct oscillation modes along the longitudinal and transverse directions of metallic nanorods have been observed to depend on the electron-beam position [49]. Furthermore, surface plasmons in Na_x_WO_3_ nanocubes have been observed under aloof-beam conditions, revealing different plasmonic modes associated with the cube corners, edges, and faces [37]. These studies indicate that this technique enables the examination of anisotropy associated with the crystal structure of individual nanoparticles with diameters of several tens of nanometers.

**Fig. 5.**
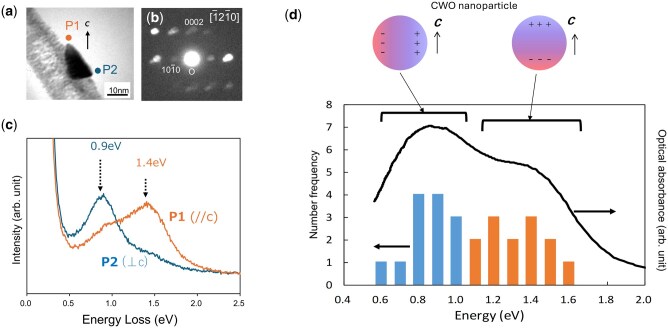
(a) TEM image of a CWO nanoparticle, and (b) crystallographic orientation of the CWO particle determined by electron diffraction. During the spectral measurements, the electron probe was placed at Position 1 (P1) or Position 2 (P2) in panel (a) for detecting plasmon oscillations parallel (//*c*) and perpendicular (⊥*c*) to the *c*-axis, respectively. (c) Resulting EELS spectra when the electron probe is placed at P1 (orange) and P2 (blue). (d) Frequency distributions of surface plasmon energies of 13 particles measured along the //*c* and ⊥*c* directions. The frequency distribution well agrees with the intensity distribution of optical scattering spectra (solid line).

Figure 5a shows the probe positions (P1: // *c* and P2: ⊥*c*) on a single CWO particle. The corresponding EELS spectra are presented in Fig. 5c. Unlike the spectrum of bulk CWO, the EELS spectra of the nanoparticles are dominated by surface plasmons with peaks at 0.9 eV (P2) and 1.4 eV (P1). The higher-energy peak at P1 indicates dipole oscillations along the *c*-axis, and the lower-energy peak at P2 corresponds to oscillations perpendicular to the *c*-axis direction. Aloof-beam EELS measurements on 13 particles yielded a frequency distribution of surface plasmon energies along // *c* and ⊥*c* (Fig. 5d). The distribution closely matched the intensity distribution of the optical scattering spectra, demonstrating that the observed NIR-light scattering originates from the anisotropic surface plasmons of CWO nanoparticles.

The high performance of CWO as a NIR-light-absorbing filter is likely sourced from the anisotropy of the plasmon energies in both bulk crystals and nanoparticles. In the filter where the CWO nanocrystals are dispersed with random crystallographic orientations, excitation of the surface plasmons along both the *c*-axis and *ab*-plane depends on the light polarization. Like the bulk plasmons, the surface plasmons oscillate at different energies along the *c*-axis and the *ab*-plane. The coexistence of these two oscillation modes across the NIR region leads to broadband scattering, which enhances the optical filtering performance.

In this study, the discussion has focused on the anisotropy of plasmons in hexagonal-structured CWO. Meanwhile, oxygen vacancies inherent to WO_3_ give rise to in-gap states, and polarons—formed when charge carriers are trapped by lattice distortions associated with these defects—also sensitively influence the optical properties in the NIR region. Machida and Adachi [35] reported that the two absorption peaks observed in the NIR region for CWO nanoparticles are significantly enhanced upon reduction treatment. Such an optical response cannot be anticipated from the dielectric function of bulk CWO, suggesting that dielectric properties are modified by nanoscale structuring. Furthermore, Yoshio *et al.* employed first-principle calculations to predict that the introduction of oxygen vacancies induces absorption peaks in the NIR region and leads to the formation of localized states (polarons) capable of trapping charge carriers. They also demonstrated that the absorption intensity at 1.6 eV originates from transitions involving these localized states, indicating that the incorporation of oxygen vacancies into the crystal can serve as a key factor in controlling NIR light-scattering performance [50]. Thus, tungsten bronze materials, including CWO, are attractive systems in which optical properties can be tuned by controlling a variety of factors, such as particle size, particle aspect ratio, carrier doping level, crystallographic anisotropy and defect introduction. To elucidate the origins of these optical properties, EELS measurements provide a highly effective analytical approach.

### XC effects of carrier plasmons in LaB_6_ crystals

The FEG model, which adopts the Lindhard dielectric function as the dielectric response [51], fundamentally describes the behavior of free electrons in a metal. However, the FEG model does not account for inter-electron exchange and Coulomb interactions (the so-called XC effects [52]). The XC effects influence the optical properties of metallic nanoparticles, including their surface plasmons resonances [53, 54]. Although this deviation is often regarded as minor in the context of light-scattering phenomena, a shift as small as 0.1 eV in the surface plasmon energy can nonnegligibly alter the color of practical optical filter materials. Therefore, the XC effects must be analyzed on a finer level than the FEG model, followed by experimental evaluations.

The XC effects can be experimentally probed with inelastic X-ray scattering [55], angle-resolved Raman scattering [56] or *q*-EELS [57–60]. The plasmon oscillations of conduction electrons are also influenced by solid-state effects such as the effective mass and interband dielectric screening [40]. Dielectric responses have been simulated within the framework of linear-response time-dependent density functional theory, which incorporates the XC effects as an approximate term describing the high-temperature, high-density plasma states (warm dense matter state) generated under intense laser irradiation [61, 62]. Experimental evaluations are crucial for assessing the reliability of these theoretical XC approximations.

The XC effects are dominant for free electrons with charge densities lower than ∼10^2^ e^-^/nm^3^ [40]. The Lindhard dielectric function must be corrected to describe the dielectric properties of such metal materials [63, 64]. The XC effects influence the plasmon *q* dispersion of an electron gas, shifting the plasmon energy to lower energies than those predicted by the FEG model with increasing *q*. Therefore, the XC effects can be quantitatively assessed from the deviation of plasmon *q* dispersion from the FEG predictions.

The XC effects can be quantified by measuring and analyzing the *q* dispersion of carrier electron plasmons in LaB_6_ crystals. Like CWO, LaB_6_ is a heat-shielding material and the mechanism of NIR-light scattering in LaB_6_ nanoparticles has attracted considerable interest. Previous studies attempted to predict the surface plasmon response of LaB_6_ particles by comparing the dielectric function of bulk LaB_6_ with NIR scattering spectra, but sufficient agreement was not achieved [65–67]. Therefore, in this study, the contribution of the XC effects on carrier electrons was clarified through an experimental evaluation.

**Fig. 6.**
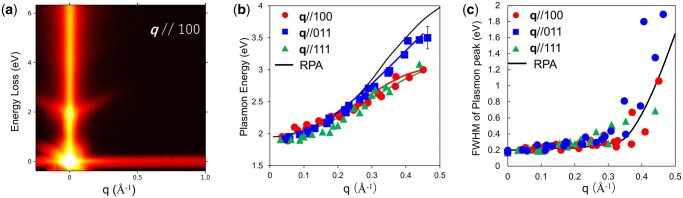
(a) *E–q* map of LaB_6_ along the ***q*** // 100 direction; *q*-dependences of (b) plasmon energy and (c) plasmon peak width. The solid lines in (b) and (c) are the predictions of the free electron gas model.

Figure 6a shows the *E–q* map of a LaB_6_ single crystal along the ***q*** // 100 direction. The spectral feature around 2 eV, originating from the carrier plasmons, disperses approximately parabolically with increasing *q*. Figure 6b summarizes the plasmon dispersions along the ***q*** // 100, 011, and 111 directions, together with the FEG-based plasmon dispersion in LaB_6_ calculated under the RPA, where the effective mass of carrier electrons in LaB_6_ is m*/m_0_ = 0.7 [68, 69] and the background dielectric function is $\varepsilon_{\infty }=7$ [27]. When *q *> 0.2 Å^−1^, the experimentally acquired plasmon dispersion lies at lower energies than the RPA prediction in all directions. The dispersion coefficients (Table 1) were obtained by fitting Eq. (7) to the experimental plasmon dispersion. The coefficient *α* clearly differs from the RPA prediction (0.86) along the ***q*** // 100 and 111 directions but more closely approaches the RPA prediction along ***q*** // 011.

**Table 1.** Coefficients of plasmon dispersion obtained by fitting *E*_P_(*q*) = *E*_P_(0) + *Aq*^2^ + O*q*^4^ to the experimental dispersions (reproduced from [27])

*q* ^a^	*Α*	*α* ^b^
100	8.3	0.76
011	10.2	0.93
111	7.2	0.66

a From the experimental values at *q* ∼0.05 Å^−1^, *E*_P_(0) was set to 1.95 eV in all *q* directions.

b Derived from the fitted coefficients *A*.

Figure 6c plots the full-widths-at-half maxima (FWHMs) of the plasmon peaks along different *q* directions. The simulated FWHM values (solid line) are based on the Mermin dielectric function [70], which incorporates a damping factor into the RPA. From the carrier plasmon FWHM at *q* ∼ 0.05 Å^−1^, the damping factor was evaluated as 0.2 eV. FWHM gradually increases in the range 0 < *q* ≤ 0.3 Å^−1^, then rises sharply at *q *> 0.3 Å^−1^ (Fig. 6c). Beyond *q* ∼ 0.4 Å^−1^, the broadening plasmon peak is no longer distinguishable, consistent with the critical value *q*_c_ = 0.39 Å^−1^ predicted by the RPA. In contrast, along ***q*** // 011, the FWHM exceeds those along the other directions and the RPA predictions at *q *> 0.35 Å^−1^.

**Fig. 7.**
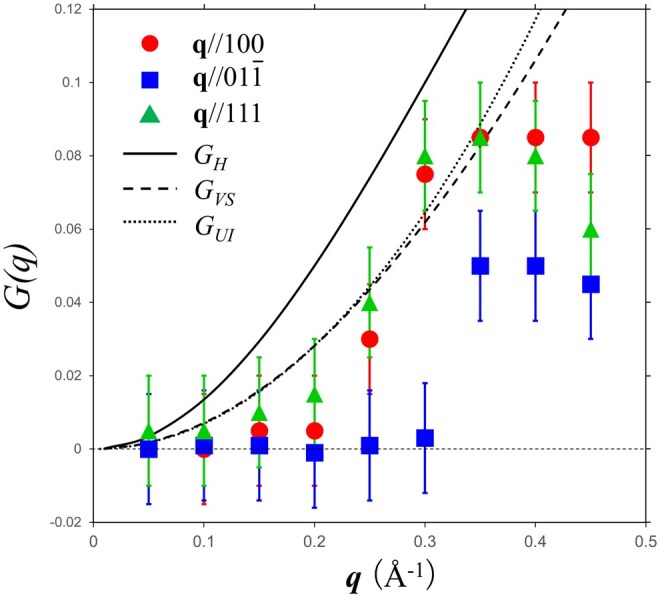
LFCs evaluated from the deviations of plasmon dispersion from the ideal free electron gas model. Three theoretical LFC distributions, G_H_ [71], G_VS_ [72] and G_UI_ [73], are shown for comparison. Reprint from [27].

The experimental results enabled a quantitative evaluation of the XC effects. The dielectric function of the quasi-FEG incorporating the XC correction is expressed as [30, 64]


(10) $$\varepsilon_{\mathrm{XC}}(q,\;\omega )=\varepsilon_{\infty }-\frac{v_{q}\chi^{0}(q,\omega )}{1+G(q)v_{q}\chi^{0}(q,\omega )},$$

where *G*(***q***) is called the local field correction (LFC). In an electron gas with a nonnegligible XC effects, the probability of finding other electrons within a short distance of a given electron is substantially lower than the FEG-predicted probability. Consequently, one can assume that each electron is surrounded by an effective positive potential. The LFC *G*(*q*) is obtained by Fourier-transforming the probability distribution [64]. The *G*(***q***) values in Fig. 7 were obtained by fitting the experimental plasmon dispersion relations to the resonance condition $\varepsilon_{\mathrm{XC}}(q,\omega )=0$.

All *G*(*q*) values were finite, indicating that the XC effects act on carrier electrons. The LFCs along ***q*** // 100 and 111 were finite, whereas that along ***q*** // 011 was nearly zero in the range 0.05 ≤ *q *≤ 0.3 Å^−1^ and remained smaller than those along the other directions up to *q *= 0.45 Å^−1^, revealing an anisotropic XC effects in LaB_6_. The experimental LFCs were compared with those of three theoretical calculations: Hubbard (G_H_) [71], Vashishta & Singwi (G_VS_) [72], and Utsumi & Ichimaru (G_UI_) [73]. The G_H_ model accounts only for exchange effects, G_VS_ includes both exchange and correlation, and G_UI_ is based on Monte Carlo calculations. As shown in Fig. 7, the LFC results along ***q*** // 100 and 111 are consistent with G_VS_ and G_UI_ at *q *< 0.35 Å^−1^, but that along ***q*** // 011 (nearly zero for 0.05 ≤ *q *≤ 0.3 Å^−1^) is significantly lower than both G_VS_ and G_UI_ at *q *> 0.3 Å^−1^. This near-zero G value suggests that despite the relatively low carrier electron density in LaB_6_, the electrons behave like a FEG along the ***q*** // 110 direction.

The behavior of FEG should be isotropic. Therefore, the anisotropic LFC profile is attributed to solid-state effects, specifically, to dielectric screening caused by interband transitions. This interpretation is supported by the FWHM broadening of the plasmon peak, which occurs at smaller *q* along ***q*** // 011 than along the other directions (Fig. 6c). The LaB_6_ band is flat with an energy separation of ∼2 eV between the valence and conduction bands along the Γ–M direction (parallel to ***q*** // 011) [74]. This flat band enables interband transitions with strong oscillator strength in the *q* ∼ 0.3–0.5 Å^−1^ range, which shift the plasmon resonances toward higher energies (2.5–3 eV). Previous dielectric analyses have approximated the interband dielectric screening constant (ε_∞_) as isotropic, but the present results suggest a nonnegligible anisotropic dependence in the region *q *> 0.3 Å^−1^.

The derived LFC demonstrates that XC effects in the carrier electrons of LaB_6_ also influence the optical properties of LaB_6_ nanoparticles used in NIR shielding filters. Specifically, under NIR-light irradiation, the induced charge density on LaB_6_ nanoparticles is reduced from that predicted by RPA, leading to a downward shift of the dipolar surface plasmon mode [53, 54]. Therefore, incorporating the XC effects is essential for accurately interpreting the optical scattering spectra of LaB_6_ nanoparticles. In the present study, literature values are adopted for the effective mass and background dielectric function of LaB_6_, as it is a well-studied material. Recent study has shown that the effective mass, background dielectric function, and Fermi velocity can be quantitatively determined from the plasmon energy and the plasmon *q*-dispersion coefficients [75]. In view of recent progress in this field, *q*-EELS has emerged as an increasingly important and comprehensive experimental method for probing the dielectric response of materials.

### Average-distance evaluation between excited electron–holes in anatase TiO_2_ using *q*-EELS

Thus far, the anisotropic optical properties and characteristics of the carrier electrons in plasmonic materials have been derived from the *q* anisotropy of the plasmon dispersion. The optical properties of such materials can be evaluated by just observing the EELS spectra. In contrast, when assessing optical functionalities arising from one-electron excitations, such as interband transitions in semiconductors, one must derive the imaginary part of the dielectric function ε_2_, which corresponds to optical absorption. The dielectric function *ε(****q****,ω)* can be derived from the *q*-EELS spectra, enabling evaluations of the *q* dependence of one-electron excitations that cannot be obtained from optical measurements.

Chemical reactions in photocatalytic materials are promoted by light irradiation. The photoexcited electron–hole pairs (excitons) migrate to the surface, where they induce redox reactions with adsorbed molecules [76]. Numerous studies have investigated the mechanisms of photocatalysis, focusing on their exciton properties such as lifetime, diffusion length and mobility [77–79]. Anatase TiO_2_ is particularly well known as a highly active photocatalyst, with longer exciton lifetimes and diffusion lengths than rutile TiO_2_. Because these properties enhance the probability of excitons reaching the material surface, they are considered to underlie the high photocatalytic activity of anatase. In addition to these properties, the spatial spread size of excitons should also influence the probability of reaching the material surface and is an important factor for understanding photocatalytic performance. Hence, correlating the spatial spread of experimental excitons with photocatalytic performance is important for understanding photocatalytic functionality.

Exciton behavior in anatase TiO_2_ has been investigated using polarization-dependent optical measurements [80]. Baldini *et al.* [81] reported that exciton-related peak structures appear in the imaginary part of the dielectric function (ε_2_) depending on the polarization directions. Specifically, under polarization perpendicular to the *c*-axis (*E_⊥c_*), absorption peaks were observed at 3.79 eV below the optical band gap and at 4.61 eV above the gap, whereas a distinct peak emerged at 4.13 eV for polarization parallel to the *c*-axis (*E_∥c_*). The energy positions and intensities of these polarization-dependent absorption peaks were well reproduced by Bethe–Salpeter equation calculations that explicitly include electron–hole interactions, supporting the assignment of these features to excitonic states. In the literature, the peaks observed at 3.79 and 4.13 eV for *E_⊥c_* are referred to as exciton-I and exciton-II, respectively, while the absorption peak at 4.61 eV for *E_∥c_* is denoted as exciton-III. Furthermore, first-principles calculations of the electron distribution associated with these excitonic states predicted that exciton-I exhibits a two-dimensional character extended within the *ab*-plane, exciton-II corresponds to a delocalized state, and exciton-III is a localized exciton with an estimated diameter of approximately 1.4 nm. These results indicate that anatase TiO_2_ hosts anisotropic excitonic states whose spatial extent strongly depends on the polarization direction. On the other hand, the spatial extent and localization size of these excitons have so far been discussed only in theoretical calculations, and direct experimental verification remains limited. Elucidating the anisotropic spatial distribution of excitons and its correlation with crystallographic facets exhibiting photocatalytic activity is therefore an important issue for advancing the understanding of the role of excitons in photocatalytic reactions.

*q*-EELS-based studies have estimated the spatial spreads of excitons in organic molecular crystals [82–84], where the spatial spreads of the excitons were derived by the *q* dependence of the absorption intensities (ε_2_) attributed to the excitonic states. This method can evaluate not only the excitons associated with dipole-allowed transitions but also the higher-order multipole excitations activated at larger *q* values. This approach has been applied exclusively to molecular crystals, where the derived exciton sizes correlate with molecular dimensions, demonstrating the validity of the method. However, its application to inorganic materials has remained unexplored. Extending this approach to the inorganic material anatase TiO_2_ enables direct evaluation of exciton spatial extent and provides insight into its correlation with photocatalytic performance.

**Fig. 8.**
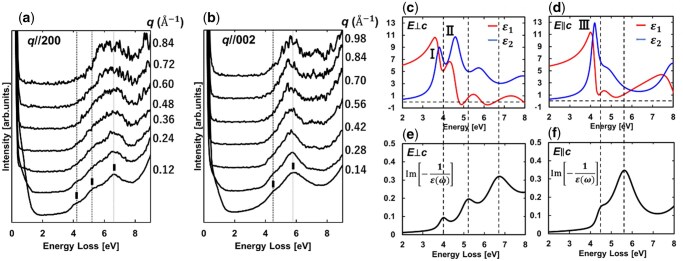
Momentum dependence of the EELS spectra of anatase TiO_2_ along (a) ***q*** // 200 and (b) ***q*** // 002; dielectric functions of polarizations (c) *E_⊥c_* and (d) *E_∥c_* derived from optical measurements [81]; intensity profiles of the loss functions calculated from the dielectric functions of (e) *E_⊥c_* and (f) *E_∥c_*. The calculated profiles well agree with the intensity profiles of the EELS spectra in (a) and (b). Reproduced from [28].

Panels (a) and (b) of Fig. 8 present the *q*-EELS spectra of anatase TiO_2_ along the ***q*** // 200 and 002 directions, respectively, in the range 0–9 eV. The spectral intensity distributions differ between ***q*** // 200 and 002, confirming an anisotropic spectral intensity distribution. Panels(c) and (d) of Fig. 8 show the dielectric functions of anatase TiO_2_ for *E_⊥c_* and *E_‖c_* polarizations, respectively, simulated by the Lorentz model to reproduce the dielectric functions derived by optical experiments [80, 81, 85]. The peaks I, II and III of ε_2_ indicated in Fig. 8c and d correspond to the exciton-I, -II and -III characteristic to anatase TiO_2_ reported by Baldini *et al.* [81]. The loss functions for *E_⊥c_* and *E_‖c_* calculated from (c) and (d) are shown in panels (e) and (f), respectively. The loss functions for *E_⊥c_* and *E_‖c_* well agree with the experimental EELS spectrum along ***q*** // 200 at *q *= 0.12 Å^−1^ and ***q*** // 002 at *q *= 0.14 Å^−1^. As demonstrated in these results, the anisotropic intensity distributions in EELS spectra reflect the excitonic absorption features contained in the imaginary part of the dielectric function.

To evaluate the spatial spread size of the excitons, the dielectric functions of anatase TiO_2_ were derived from the EELS spectra at various *q* values through a Kramers–Kronig analysis, in which the intensity of the zero-loss peak tail was subtracted using Lorentz fitting. The multiple-scattering intensity was removed via Fourier-log deconvolution, yielding the single-scattering distribution of each experimental spectrum [86]. Applying the sum rule, the absolute value of the loss function was obtained as


(11) $$\int_{0}^{\infty }\omega \text{Im}[-\frac{1}{\varepsilon (\omega ,q)}]d\omega =\frac{\pi }{2}\omega_{p}^{2},$$

where $\omega_{p}$ is the volume plasmon frequency, estimated as 7.54 × 10^15^ rad/s from the valence electron density of anatase TiO_2_ [28]. The experimental EELS spectra were measured in the energy region up to 90 eV and extrapolated to energies above 90 eV using the equation ${aE}^{-b}$ fitted from 85 to 90 eV, where a and b are the fitting parameters, and E is the energy. Dielectric functions at each *q* were derived through the Kramers–Kronig relation. The integrations over energy for the sum rule and the Kramers–Kronig relation were conducted up to 400 eV.

**Fig. 9.**
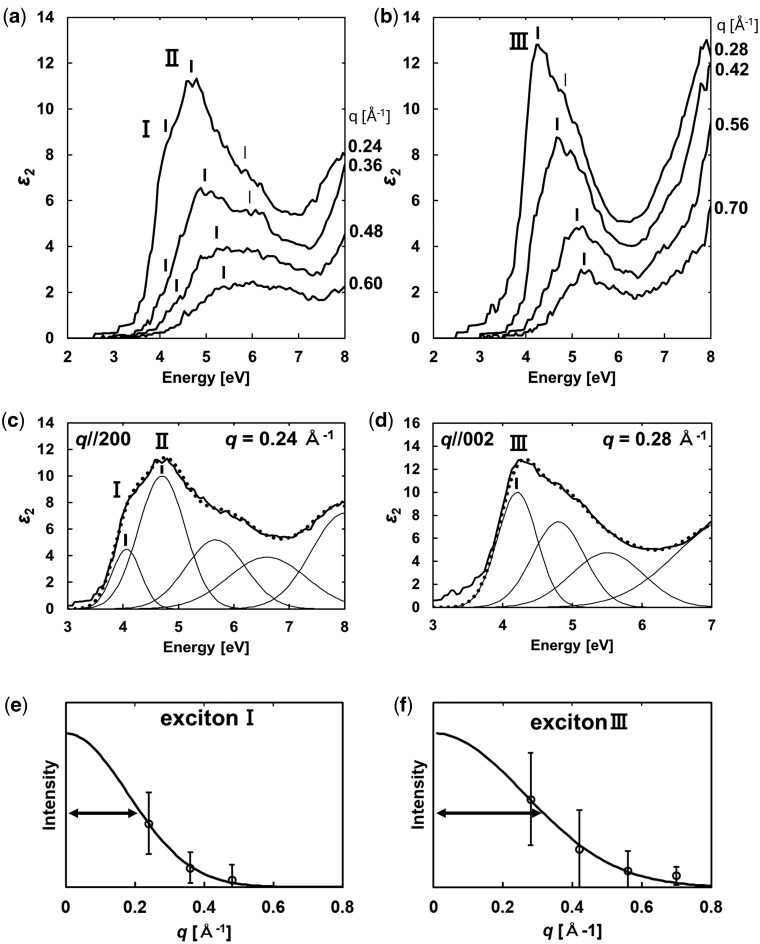
Momentum dependences of the imaginary part of the dielectric function (*ε*_2_) of anatase TiO_2_ along the (a) ***q*** // 200 and (b) ***q*** // 002 directions. Spectral features I–III correspond to excitons I and II. Intensities of exciton peaks (c) I and (d) III, quantitatively obtained by fitting the imaginary part of the dielectric function. Momentum dependences of the peak intensities of (e) exciton I and (f) exciton III. The solid curves are Gaussian fittings. The inverse of the full width at half maximum was taken as the real-space extension of the excitons.

Figure 9 shows the imaginary part of the dielectric function ε_2_ of anatase TiO_2_ derived from the EELS spectra. The spectral structure I and peak structure II in Fig. 9a correspond to the exciton-I and -II of *E_⊥c_* in Fig. 8c, respectively, and the peak in Fig. 9b coincides with exciton-III of *E_∥c_* in Fig. 8d, demonstrating consistency between the dielectric functions in the present analysis and those derived from optical experiments. To numerically estimate the intensities of the bound exciton-I and -III, Gaussian fittings were applied along ***q*** // 200 and ***q*** // 002, respectively (Fig. 9c and d). In these fittings, the energy positions of the spectral features were fixed while the intensity and peak width were treated as fitting parameters. Figure 9e and f displays the *q*-dependent absorption intensity variations of excitons I and III, respectively. In both cases, the intensities decrease with increasing *q*, as observed for dipole transitions in the matrix elements of inelastic scattering [82], indicating optically allowed transitions. The absorption intensity profiles were also fitted with Gaussian functions, taking the inverse of the half-width as a measure of the spatial exciton extent in real space. As the results, the sizes of excitons I and III were estimated as 8 (±2) and 6 (±2) nm, respectively.

According to first-principles calculations, exciton-I is distributed within the *ab*-plane with an approximate diameter of 6.4 nm and is localized along the *c*-axis, forming a two-dimensional distribution [81]. In contrast, exciton-III is predicted to be isotropically localized with a diameter of ∼1.4 nm. In the present study, the evaluated 8 nm extent of exciton-I corresponds to its spread along the *a*-axis, which reasonably agrees with theoretical prediction. Similarly, the evaluated 6 nm extent of exciton-III represents its spread along the *c*-axis. Although the experimental value of exciton-III exceeds the theoretical prediction, the smaller spread size of exciton-III than of exciton-I is consistent with the overall theoretically predicted trend. Therefore, the *q*-EELS-based analysis can effectively estimate the spatial spreads of excitons.

The photocatalytic activity of anatase TiO_2_ reportedly depends on the crystal plane, being highest on the (100) plane and lowest on the (001) plane during hydroxylation of terephthalic acid [87]. This facet-dependent activity is closely related to the two-dimensional spatial spread of exciton-I in anatase TiO_2_. Being delocalized within the *ab*-plane, this exciton can readily reach the (100) plane but has limited access to the (001) plane, correlating with the photocatalytically active surfaces. These findings suggest that exciton-I plays a crucial role in governing the photocatalytic activity of anatase TiO_2_; moreover, the real-space diffusion of excitons largely determines the photocatalytic performance. In addition, amorphous TiO_2_ has been reported to exhibit excellent photocatalytic properties [88, 89]. An analytical approach based on *q*-EELS has been proposed for evaluating exciton sizes in amorphous solids using amorphous SiO_2_ as a model system [90]. Future studies should aim to comprehensively evaluate the lifetime, diffusion length and spatial extent of excitons in various photocatalytic materials. Such studies would clarify the origin of the functional properties of excitons and guide the design of higher-performance materials.

## Conclusion

This review presents several examples of *q*-EELS measurements and analyses of photo-functional materials using monochromated TEM. Momentum-resolved measurements enable evaluations of anisotropic optical properties, electron–electron interactions (the XC effects; not considered in the FEG model) and exciton spatial spread size, providing deeper insights into the origin of photo-functionality. Furthermore, as *q*-EELS analysis advances, it is expected to reveal the *q* dependence of interband transitions reflecting band dispersions, providing valuable information on electronic band structures. Thus, further development of *q*-EELS methodology will not only promote the design of high-performance photo-functional materials but also deepen our understanding of electronic structures in a wide range of solids.

## Data Availability

The data supporting the findings of this study are available from the corresponding author upon reasonable request.
